# Anisotropy of Single-Crystal Silicon in Nanometric Cutting

**DOI:** 10.1186/s11671-017-2046-4

**Published:** 2017-04-26

**Authors:** Zhiguo Wang, Jiaxuan Chen, Guilian Wang, Qingshun Bai, Yingchun Liang

**Affiliations:** 1 School of Mechatronics Engineering, Harbin Institute of Technology, Harbin, 150001 People’s Republic of China; 2 College of Mechanical Engineering, Tianjin University of Technology, Tianjin, 300384 People’s Republic of China

**Keywords:** Molecular dynamics simulation, Nanometric cutting, Single-crystal silicon, Anisotropy behavior, Subsurface damage, Chip

## Abstract

The anisotropy exhibited by single-crystal silicon in nanometric cutting is very significant. In order to profoundly understand the effect of crystal anisotropy on cutting behaviors, a large-scale molecular dynamics model was conducted to simulate the nanometric cutting of single-crystal silicon in the (100)[0–10], (100)[0-1-1], (110)[−110], (110)[00–1], (111)[−101], and (111)[−12-1] crystal directions in this study. The simulation results show the variations of different degrees in chip, subsurface damage, cutting force, and friction coefficient with changes in crystal plane and crystal direction. Shear deformation is the formation mechanism of subsurface damage, and the direction and complexity it forms are the primary causes that result in the anisotropy of subsurface damage. Structurally, chips could be classified into completely amorphous ones and incompletely amorphous ones containing a few crystallites. The formation mechanism of the former is high-pressure phase transformation, while the latter is obtained under the combined action of high-pressure phase transformation and cleavage. Based on an analysis of the material removal mode, it can be found that compared with the other crystal direction on the same crystal plane, the (100)[0–10], (110)[−110], and (111)[−101] directions are more suitable for ductile cutting.

## Background

Single-crystal silicon is an essential material for fabrication of many kinds of components in optoelectronics, infrared optics, microelectronics and micro electro-mechanical systems (MEMS) [1, 2], etc. With the rapid development of these industries in recent years, the demand for silicon materials has grown exponentially. Therefore, it is extremely necessary to quickly and efficiently fabricate silicon components and parts with perfect surface integrity. Compared with the traditional processing technologies of silicon, such as grinding, lapping, etc., the nanometric cutting technology is not only able to directly manufacture highly accurate machined surfaces with excellent surface roughness and crack free but also to make complex surface features on silicon wafers [3, 4], which could be widely applied in the future. However, single-crystal silicon is of prominent anisotropy, and obvious differences appear in its physical and mechanical properties, such as hardness, elasticity modulus, yield limit and phase transformation pressure, etc., with changes in crystal plane and crystal direction [5–8]. Thus, the surface integrity of silicon wafer shows apparent anisotropy during nanometric cutting [7], which differs the performance of silicon components with change in crystal direction under work condition [6, 9]. Hence, it is significant to study the impact of silicon anisotropy on its cutting behaviors in nanometric cutting.

During the single-point diamond turning of silicon (100) and (111) crystal planes, Shibata et al. [10] found that visible differences occurred in machined surface quality with changes in machining surface and cutting direction. In order to explain the anisotropy of the surface quality, Wang et al. [7] presented a numerical simulation model that effectively connected crystal structure and mechanical property and validated the simulation result through experiments. On the basis of the nanometric cutting experiments on silicon, Yan et al. [11] reported that the thickness and structure of the subsurface damage (SSD) had a close relationship with the cutting direction. As an effective supplementary means to experimental study, molecular dynamics (MD) simulation was also applied to the research on anisotropy of crystalline materials. Komanduri et al. [12] analyzed the impact of anisotropy in single-crystal aluminum, which is a kind of plastic material, on material removal and subsurface deformation mechanisms. In addition, Lai et al. [13, 14] studied the anisotropic behaviors in single-crystal germanium (a brittle material) exhibited during nanometric cutting and nanoindentation. Goel et al. [15] simulated the nanometric cutting process of 3C-SiC in nine combinations of crystal planes and crystal directions. Results obtained through comparing the temperature and stress in the machining area and cutting force show that the (001)[100], (110)[001], and (111)[−110] crystal directions, relative to the other direction on the same crystal plane, were much more prone to be removed in ductile mode. Moreover, Goel et al. [16] also investigated the influence of crystal orientation on wear resistance of a diamond tool and found that cubic orientation performed better than dodecahedral orientation. Chavoshi et al. [17] studied the effect of anisotropy in silicon on chip formation mechanism.

Previous studies have provided many valuable insights into the nanometric cutting mechanisms of single-crystal silicon [17–23]. However, few reports on the silicon anisotropy exhibited during nanometric cutting resulted in the lack of deep understanding of its formation mechanism. Therefore, in this study, a large-scale MD simulation of nanometric cutting of single-crystal silicon in different crystal directions using a diamond cutting tool was performed to study the effects of crystal anisotropy on its cutting behaviors. Crystal planes (100), (110), and (111) of silicon were taken as the machining surfaces, and two typical crystal directions on each selected crystal plane were considered as the cutting directions. The mechanisms of material removal and surface damage formation in the nanometric cutting were preliminarily studied by analyzing chip, surface damage, cutting force, and friction coefficient.

## Methods

**Fig. 1:**
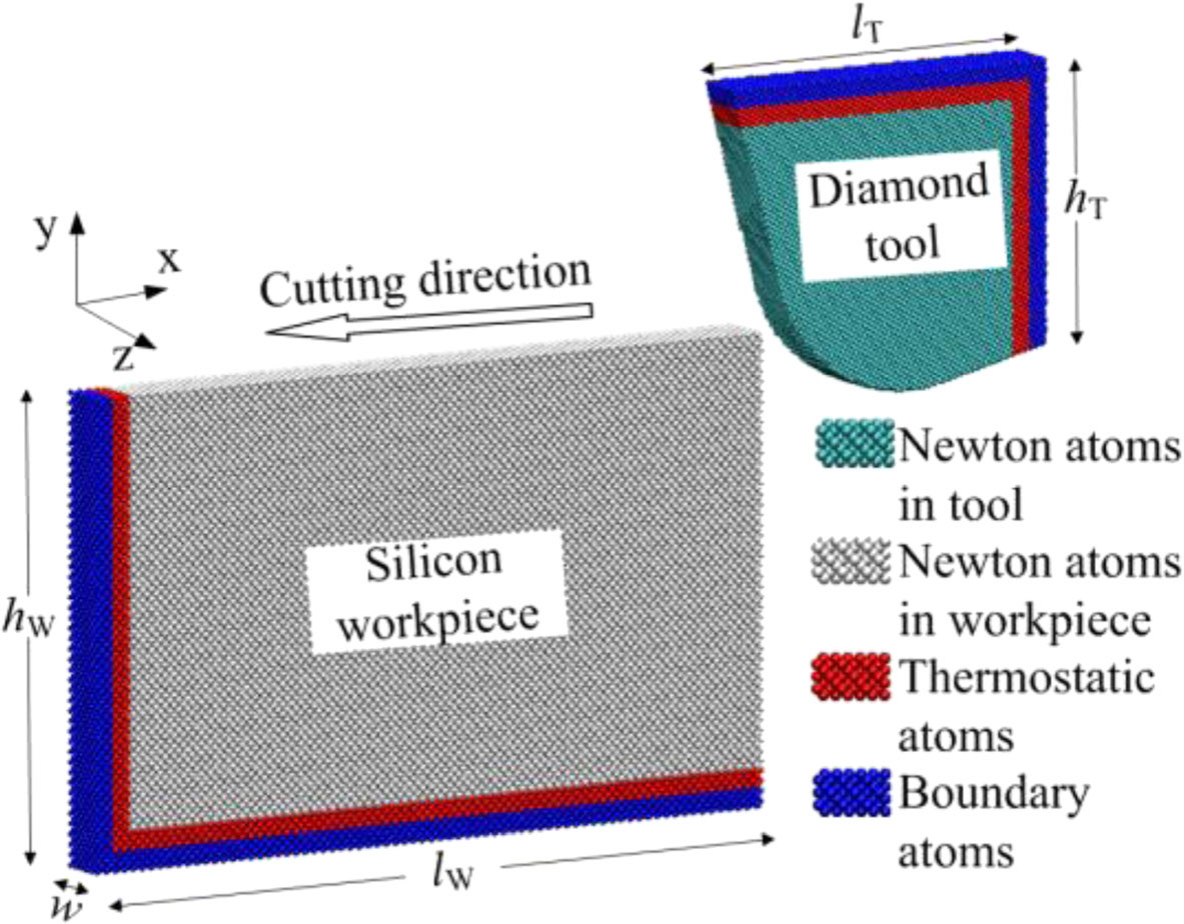
Schematic diagram of MD simulation model for nanometric cutting

Figure 1 illustrates the schematic diagram of large-scale MD simulation model for nanometric cutting of single-crystal silicon using a diamond tool. The parameters used in the simulations are shown in Table 1. In order to make the simulation closer to the actual cutting process, the silicon workpiece and the diamond tool were set as deformable body and were classified into three parts: Newton layer, thermostatic layer, and boundary layer. The Newton layer is a machining area, in which all atoms are kept within Newton’s laws of motion. The thermostatic layer is used to absorb the cutting heat delivered from the machining area, preventing any adverse effect due to high temperature on the cutting process. The boundary layer keeps the workpiece away from movement during the cutting process. Both the thermostatic layer and the boundary layer are 2-nm thicknesses. Periodic boundary conditions are adopted in the *z* direction to reduce the size effect caused by the small size in this direction.

**Table 1:** Parameters used in the simulations

Machining parameters	Value
Workpiece size (l_W_ × h_W_ × w)	100 × 50 × 3 nm^3^
Number of silicon atoms	72 × 10^4^
Tool size (l_T_ × h_T_ × w)	25 × 25 × 3 nm^3^
Number of carbon atoms	26 × 10^4^
Radius of cutting edge	8 nm
Rake and clearance angles of tool	−7° and 7°
Cutting depth	6 nm
Cutting speed	200 m/s
Cutting direction	(100)[0–10] and (100)[0-1-1] (110)[−110] and (110)[00–1] (111)[−101] and (111)[−12-1]
Temperature	293 K
Time step	2 fs

It is well known that the physical and mechanical properties of crystalline materials exhibit obvious anisotropy under different crystal planes and crystal directions. Currently, (100), (110), and (111) crystal planes are widely studied in many kinds of processing experiments and computer numerical simulations [7, 10, 24, 25]. In the MD research on nanometric cutting process of crystalline materials, <001> and <110> crystal systems for (100) and (110) planes, and <110> and <112> systems for (111) plane are mainly investigated [12, 15]. Hence, two typical crystal directions for each (100), (110), and (111) planes are separately chosen in this study, which are shown in detail as follows: [0–10] and [0-1-1] directions for (100) plane, [−110] and [00–1] directions for (110) plane, and [−101] and [−12-1] directions for (111) plane.

In this study, LAMMPS [26], which is a kind of MD software developed by Sandia National Laboratories, was adopted to simulate the nanometric cutting process of silicon. During simulation, Tersoff potential was employed to describe the interaction of C–C and Si–Si [27]. Similarly, Morse potential was adopted to depict the interplay of C–Si [28]. The whole simulation fell into two steps: first, the temperature of the thermostatic layer was kept at around 293 K by adjusting velocities of the thermostatic atoms throughout the simulation, and a relaxation of 40,000 time steps available in the system was made to ensure the temperature of the Newton layer could fluctuate slightly at the specified one and the energy could remain in a balanced state; next, the workpiece was cut by the diamond tool at a constant speed along the −*x* direction up to a 100-nm distance, and the cutting was ended.

## Results and Discussion

### Mechanisms of Nanometric Cutting

**Fig. 2:**
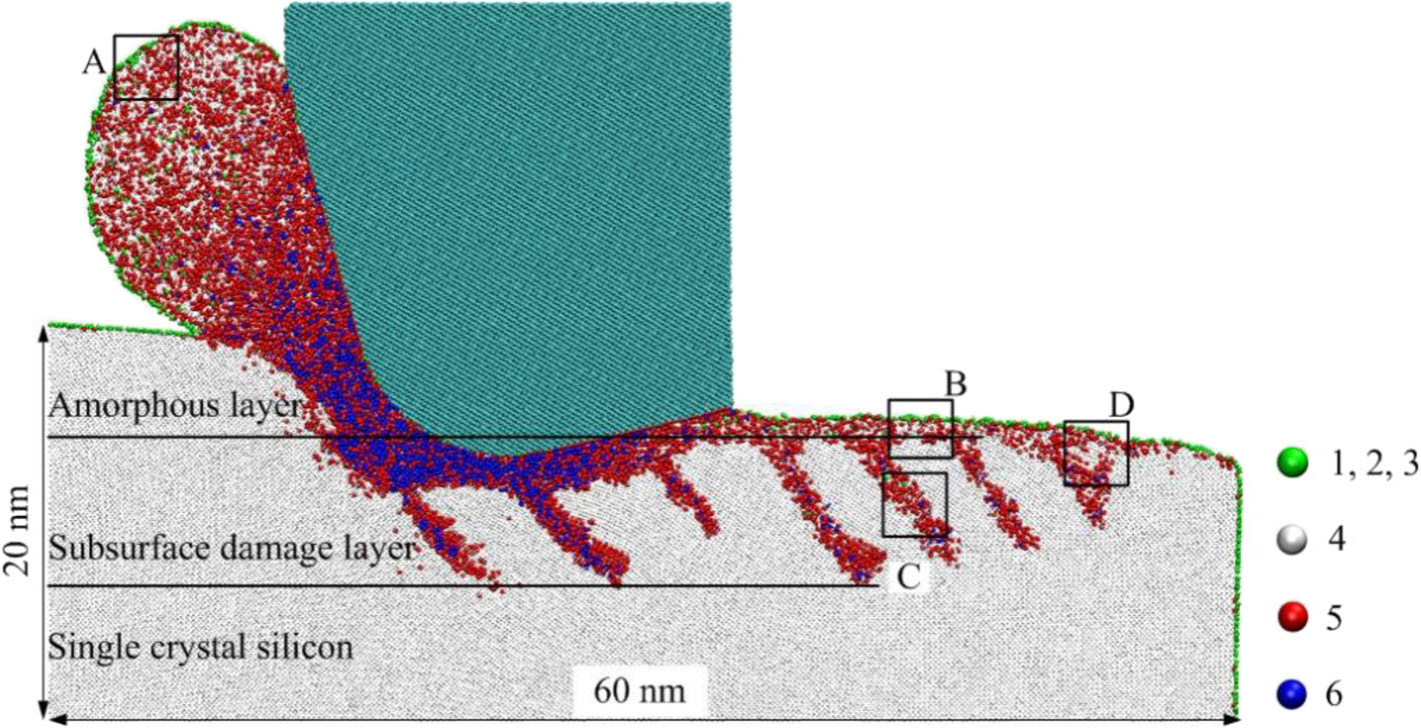
Sectional view on the (100)[0–10] crystal direction at a cutting distance of 50 nm

**Fig. 3:**
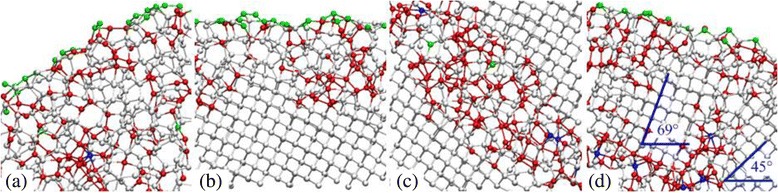
Enlarged views of **a** A zone, **b** B zone, **c** C zone, and **d** D zone in Fig. 2

Figure 2 shows the sectional view of the silicon workpiece on the (100)[0–10] crystal direction at a cutting distance of 50 nm. In this figure, the silicon atoms are colored according to their coordination numbers (CNs); green color indicates a silicon atom with low CNs of 1–3; white, red, and blue colors represent silicon atoms with CNs of 4, 5, and 6, respectively; and cyan color shows a carbon atom in a diamond tool. The silicon atom in the diamond cubic structure with a CN of 4 represents single-crystal silicon (c-Si), and the silicon atoms in the body-centered tetragonal structure with CNs of 5 and 6 are respectively bct5-Si and β-Si in MD simulations of nanometric machining [9, 29]. It can be seen that the chip formation zone consists of the silicon atoms with the CNs of 5 and 6. This indicates that the phase transformation from c-Si to bct5-Si and β-Si occurs in the chip formation zone. As the distance away from the chip root increases, the number of bct5-Si and β-Si in the chip gradually decreases, and silicon atoms with a CN of 4 increase, accordingly. From the enlarged view of the A zone in Fig. 2 and as shown in Fig. 3a, the top of the chip is mainly made up of the amorphous silicon (a-Si) with CNs of 1–5 that has a long-range disordered structure. Moreover, no crystallite that retains the diamond cubic structure can be observed in the chip. The findings above show that the silicon workpiece on the (100)[0–10] crystal direction is removed in the manner of ductile mode during the nanometric cutting process.

It is evident from Fig. 2 that the machined silicon workpiece is split into three layers of upper, middle, and lower along the *y* direction. The upper layer consists of the long-range disordered a-Si, which is a surface a-Si layer, as shown in Fig. 3b. The middle layer is a SSD layer, in which the main body is still c-Si. However, several strip-type plastic deformation zones scatter in this layer. It can be seen in Fig. 3c that the plastic deformation zone is also comprised of the long-range disordered a-Si that include plenty of atoms with CNs of 4 and 5, as well as a handful of atoms with CNs of 3 and 6. This indicates that an amorphous phase transformation occurs in the subsurface. The SSD structure obtained in this study is in complete consistence with the simulation results of Tanaka [30] and Zhao [31], and Tanaka defined this structure as shear deformation. In addition, as shown in Fig. 3d, a small microcrystal grain that retains its original crystal structure can be observed in the plastic deformation zone below the surface a-Si layer, and its lattice orientation differs by 24° from the surrounding crystalline silicon. This suggests that the SSD contains polycrystalline grains. This polycrystalline structure has been common in nanometric cutting experiments of silicon [11, 32]. Obviously, the lower layer is the c-Si layer, in which the silicon atoms continue to keep their original structure.

**Fig. 4:**
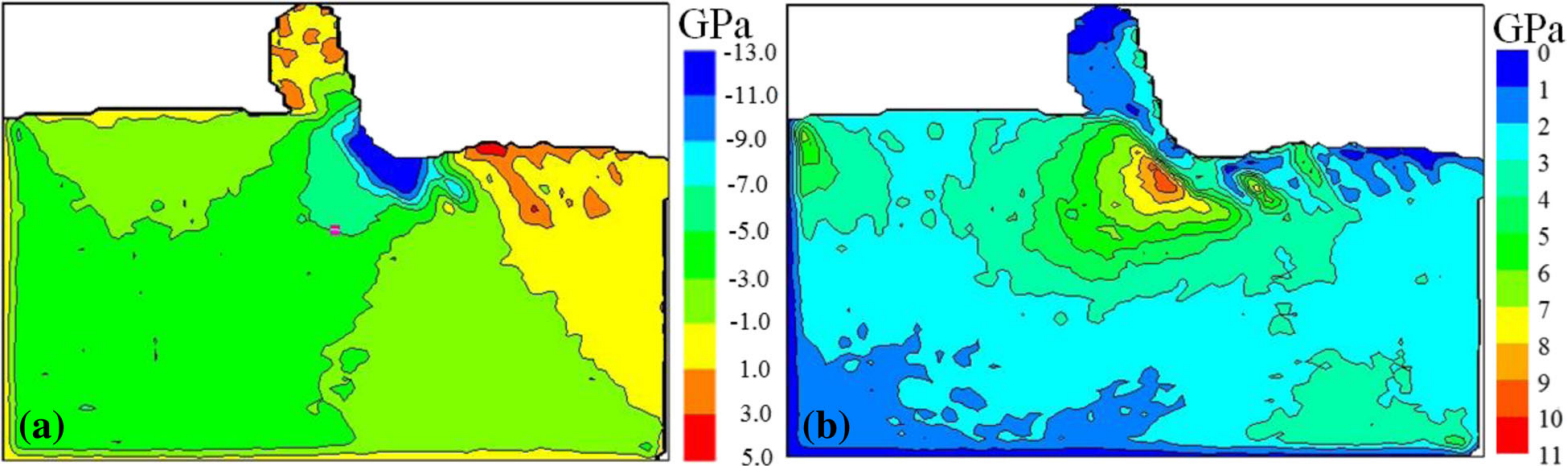
**a** Hydrostatic pressure and **b** the maximum shear stress in silicon

In this MD simulation study, hydrostatic pressure and the maximum shear stress were used as the main criterion to predict yielding of silicon during its nanometric cutting [33]. The scalar stress values were determined by converting atomic stress tensor to physical stress tensor which was then fed to the formulas shown in the “Appendix” to calculate the two kinds of stresses. Figure 4 shows hydrostatic pressure and the maximum shear stress in the silicon workpiece at the cutting distance of 50 nm. It can be shown in Fig. 4a that the highest hydrostatic pressure is located in a narrow region near the cutting edge which is about −11 to −13 GPa; the quantity does not appear to be much different from the metallic transformation pressure of c-Si (−10 to −13 GPa) obtained in the experiments [29]. This phenomenon indicates that during nanometric cutting process, c-Si near the cutting edge transforms into bct5-Si and β-Si, under the effect of high hydrostatic pressure. That is to say, the c-Si experiences a high-pressure phase transformation (HPPT). Among the bct5-Si and β-Si in the HPPT zone, one part constitutes the chip, and the other part forms the machined surface under subsequent cutting action of the diamond tool. Moreover, it can be seen that the hydrostatic pressure in the chip and the machined surface rapidly decreases to about −1–3 GPa, which further makes the bct5-Si and β-Si change into a-Si. Thus, the HPPT is considered as the formation mechanism of the completely amorphous chip and the surface amorphous layer during the ductile cutting. However, the hydrostatic pressure is below the critical pressure for the HPPT, the metallic phase transformation of c-Si in the subsurface outside the HPPT zone is restrained. As shown in Fig. 4b, the maximum shear stress in the subsurface under the diamond tool can reach as high as 9 GPa, which is very close to the theoretical shear stress of c-Si (9.23 GPa) [34], and the maximum shear stress direction is the same as the SSD direction in Fig. 2. When there is high shear stress existing in silicon wafers, it can be found that c-Si can directly transform into a-Si, even if hydrostatic pressure is below the critical pressure for the HPPT [35–37]. These results indicate that the SSD is induced by high shear stress, which proves the shear deformation mechanism during nanometric cutting of silicon proposed by Tanaka [30].

### Analysis on Removal Mechanism

**Fig. 5:**
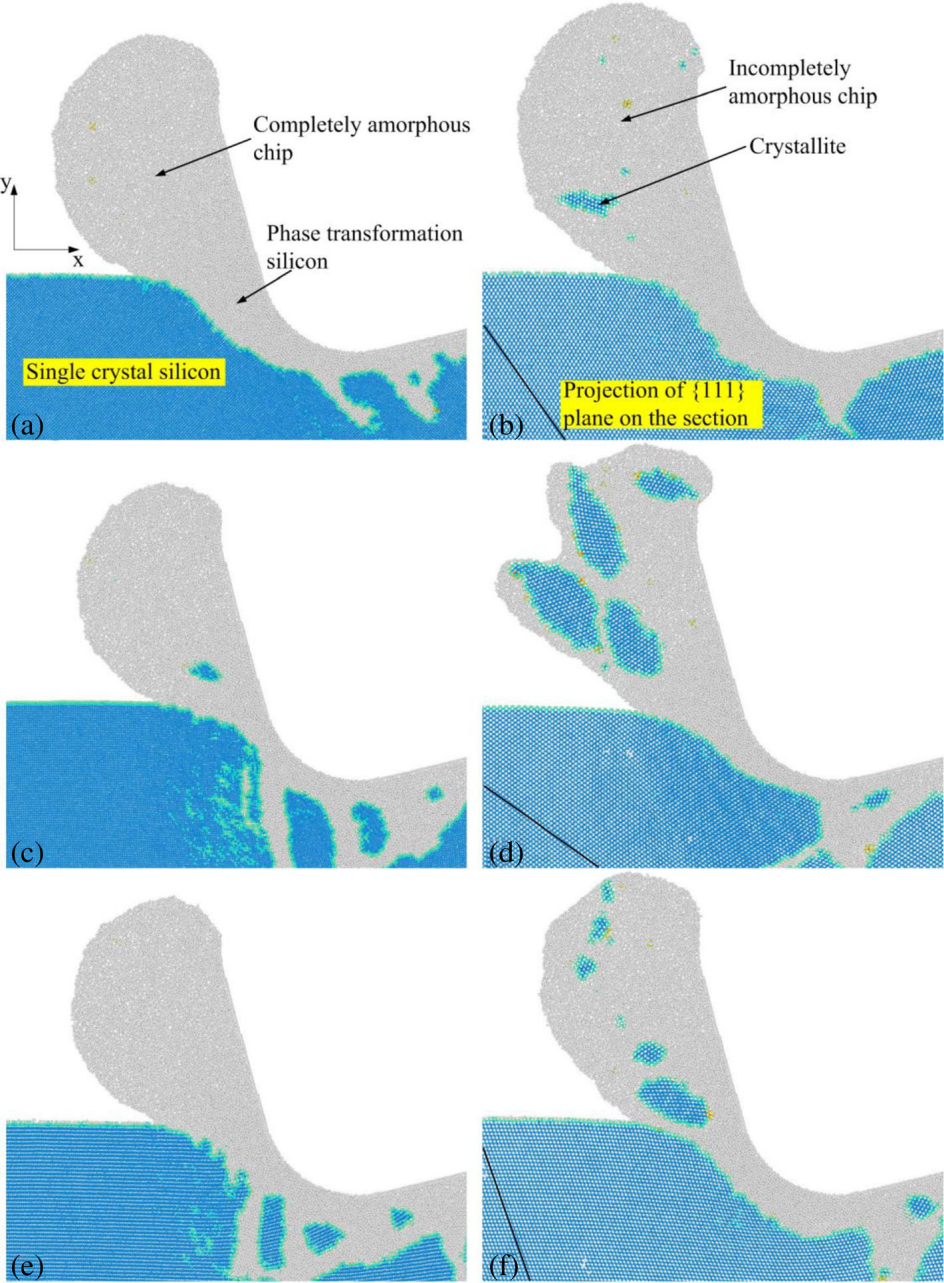
Chip structures for **a** (100)[0–10], **b** (100)[0-1-1], **c** (110)[−110], **d** (110)[00–1], **e** (111)[−101], and **f** (111)[−12-1] crystal directions at a cutting distance of 70 nm

**Fig. 6:**
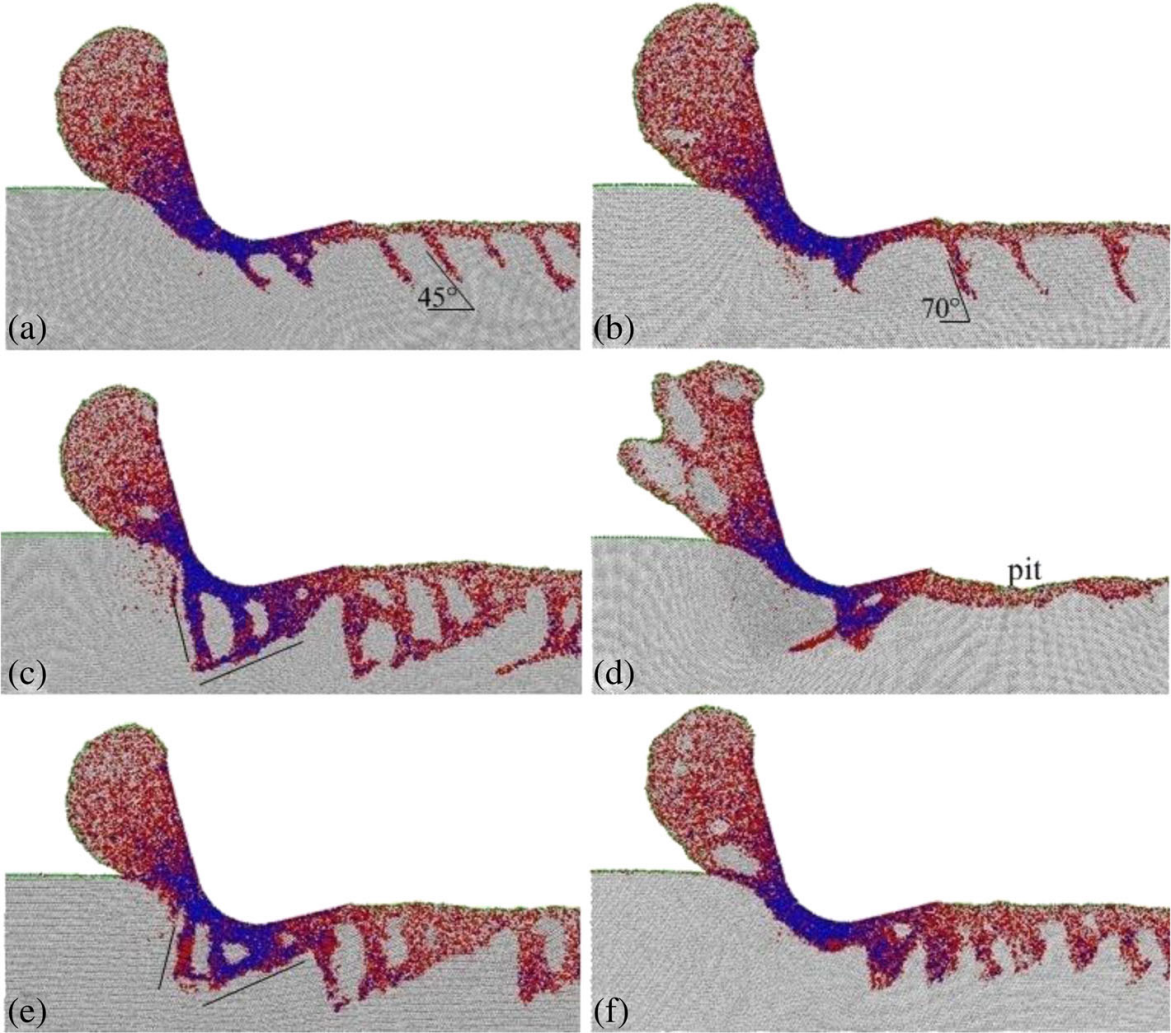
Sectional views on the **a** (100)[0–10], **b** (100)[0-1-1], **c** (110)[−110], **d** (110)[00–1], **e** (111)[−101], and **f** (111)[−12-1] crystal directions at a cutting distance of 70 nm

In order to observe the chip structures for different crystal directions clearly, a diamond structure identification method in the Ovito software [38] was adopted to identify silicon atoms with the diamond structure in the chips, as shown in Fig. 5. In the figure, light blue color represents a silicon atom with the diamond structure; white color indicates a phase transformation silicon atom that is made up of a-Si, bct5-Si, or β-Si. It is evident that six chips could fall into two different structures, in which one is a completely amorphous chip (Fig. 6a, e) and the other is an incompletely amorphous chip that includes a few crystallites (Fig. 6b–d, f). In the previous MD simulations of nanometric cutting of brittle materials, the former was common, while the latter was barely observed [13, 30, 31]. However, these two kinds of chips have been proven in the nanometric cutting experiments [21].

As shown in Fig. 5a, e, the chips on the (100)[0–10] and (111)[−101] crystal directions are totally comprised of a-Si. On account of significantly higher microplasticity of a-Si than c-Si [39, 40], the amorphous chip generates plastic flow along the rake face of diamond tool due to tool extrusion and produces a smooth appearance. However, it can be seen in Fig. 5b–d, f that the crystallites are obviously observed in the chips on the (100)[0-1-1], (110)[−110], (110)[00–1], and (111)[−12-1] crystal directions. The larger the crystallites are, the more serious the distortion on the chip is. As a result, the crystallites in the chip for the (110)[00–1] crystal direction have the largest size. Thus, the chip deforms most severely, which is similar to the brittle chips in the experiments [23].

It can be found from the chip structures in Fig. 5 that there are two kinds of material removal mechanisms in the nanometric cutting processes of silicon. When silicon is removed by HPPT, the chip obtained is the amorphous structure. Contrarily, when HPPT is difficult to occur, high shear stress in silicon in front of the diamond tool is caused by the tool extrusion. The shear stress in a cleavage plane is beyond the yield limit of c-Si, and then an amorphous transformation from the HPPT zone to the untreated surface along the cleavage plane can take place, which results in occurrence of cleavage and formation of a crystallite. These suggest that the completely amorphous chips for the (100)[0–10] and (111)[−101] directions are generated by HPPT; however, the incompletely amorphous chips for the (100)[0-1-1], (110)[−110], (110)[00–1], and (111)[−12-1] directions are formed by HPPT and cleavage. Silicon and 3C-SiC are both the diamond cubic structure, which have relatively similar physical properties and nanometric cutting behaviors. Hence, the same removal mechanisms were also proposed by Goel, when the anisotropy behaviors of 3C-SiC were investigated in nanometric cutting. c-Si has an octahedral crystal structure, where the eight {111} planes comprise the cleavage planes [7]. The cleavage plane perpendicular to the *x*-*y* section is the main one as the black line in Fig. 5b, d, f, except the machining face, the others are the non-main ones. The shear component induced by the tangential force in the main cleavage plane is higher than the non-main ones, for the projection of the main cleavage plane in *x*-*y* section is coplanar with the tangential force. That is to say, the cleavage is the easiest along the main cleavage plane. Therefore, the heavy cleavage occurs in the three crystal directions containing the main cleavage plane. However, cleavage does not happen in the directions not including the main cleavage plane, except the (110)[−110] direction. It can be concluded that the yield limit of the (110) plane is lower than the (100) and (111) planes because of the heaviest cleavage in the (110)[00–1] direction among the six crystal directions. Hence, the cleavage is also observed in the (110)[−110] direction, but it is obviously slighter than the (100)[0-1-1], (110)[00–1], and (111)[−12-1] directions.

It can be found that only the (100)[0–10] and (111)[−101] directions can be completely removed in the ductile mode among the six selected directions, according to the analysis of the material removal mode. However, compared to the ductile machinability for the both directions on the same crystal plane, the (100)[0–10], (110)[−110], and (111)[−101] crystal directions are superior to the (100)[0-1-1], (110)[00–1], and (111)[−12-1] ones, respectively.

### Analysis on Surface Damage

Figure 6 shows the sectional views of the silicon workpieces on different crystal planes and crystal directions at a cutting distance of 70 nm. It can be seen in Fig. 6 that the SSD structure obviously varies as the crystal plane and crystal direction change. As shown in Fig. 6a, the SSD on the (100)[0–10] crystal direction consists of the shear deformation in a single direction, which is large in amount and 45° from the *x*-axis. The SSD on the (100)[0-1-1] direction (Fig. 6b) is similar to the former, but its shear deformation forms 70° from the *x*-axis and is small in amount. The SSD on the (110)[−110] direction (Fig. 6c) is extremely complex and almost consists of the shear deformations along the two straight lines in the figure. Moreover, several large polycrystalline grains spread in the subsurface. The SSD on the (110)[00–1] direction (Fig. 6d) is relatively slight and only lies in the subsurface under the tool. However, the machined surface that has a 1.4-nm deep pit is visible. According to experimental results, silicon wafers removed in brittle mode would form brittle chips and leave pits on the machined surface [20, 23]. These phenomena are consistent with the simulated results for the (110)[00–1] direction, suggesting that the silicon workpiece was removed in brittle mode. The SSD on the (111)[−101] direction (Fig. 6e) is similar to that on the (110)[−110] direction. This is the result of the shear deformations of two approximate directions for these two crystal directions. Due to the lack of a main shear deformation direction, the SSD on the (111)[−12-1] direction (Fig. 6f) is shallow and wide.

Table 2 shows the machined surface integrity achieved by cutting in different crystal directions. From the table, the surface integrity was found to be obviously different as the crystal plane and crystal direction changed. Among the six crystal directions, the both directions on the (100) plane are the best two for the surface integrity, but the (100)[0–10] direction is a little poorer than the (100)[0–11] direction; the surface roughness and surface damage depth of the former are 0.53 and 9.4 nm, respectively; the (110)[−110] direction is the worst; its surface roughness and surface damage depth are 0.9 and 14.2 nm, separately. In other words, the (100) plane is of the best surface integrity, and the (110) plane is of the worst in nanometric cutting of silicon. Furthermore, it can be seen in Fig. 6 and Table 2 that the surface integrity is not close relationship with cleavage but depends on a SSD degree, though the cleavage occurs in the (100)[0-1-1], (110)[00–1] and (111)[−12-1] directions. The more serious the SSD degree is, the poorer the surface integrity is. This is because the cleavage degree is lighter due to the thinner cutting depth, which does not generate serious pits on the machined surface.

**Table 2:** Surface integrity on different crystal directions

	(100)[0–10]	(100)[0-1-1]	(110)[−110]	(110)[00–1]	(111)[−101]	(111)[−12-1]
a-Si layer thickness/nm	1.5	1.3	1.7	2	1.3	1.6
SSD layer thickness/nm	7.9	8.5	12.5	6.2	11.1	8.6
Surface roughness/nm	0.53	0.3	0.9	0.72	0.73	0.54

When cutting silicon in the (100)[0-1-1], (110)[00–1], and (111)[−12-1] crystal directions, the shear deformation in the subsurface has a smaller quantity or lighter depth, compared with the other direction on the same crystal plane, and the chips for these directions contain crystallites. These results indicate that the shear deformation is hardly formed in the subsurface of the three workpieces, resulting to their material removal through cleavage and HPPT.

### Analysis on Cutting Force and Friction Coefficient

**Fig. 7:**
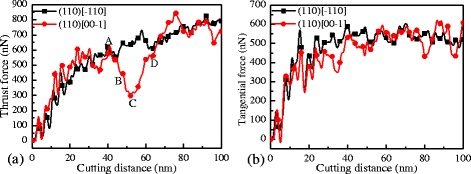
**a** Thrust forces and **b** tangential forces during nanometric cutting on the (110) crystal plane

In order to study the effect of cleavage and shear deformation on cutting force, the (110) crystal plane where the most obvious cleavage and shear deformation were observed during the nanometric cutting process is taken as an example (Fig. 7). High-frequency fluctuation in the cutting force caused by atomic thermal motion [13] is eliminated to observe the fluctuation induced by cleavage and shear deformation in detail by fitting. Tangential force and thrust force refer to the cutting forces along the *x* and *y* directions, respectively. It can be seen in the figure that both the thrust force and the tangential force have quite vigorous fluctuations irrespective of the crystal direction. The wavelengths of these fluctuations (varying from several nanometers to tens of nanometers) are close to the distances between neighboring shear deformations or neighboring cleavages. Furthermore, through careful observation, it is found that the wavelength and amplitude in the cutting forces on the [00–1] crystal direction are larger than those on the [−110] direction. This indicates that the vigorous fluctuations in the cutting forces are mainly caused by the cleavage and shear deformation, and the fluctuations caused by the former are more serious than the latter.

**Fig. 8:**
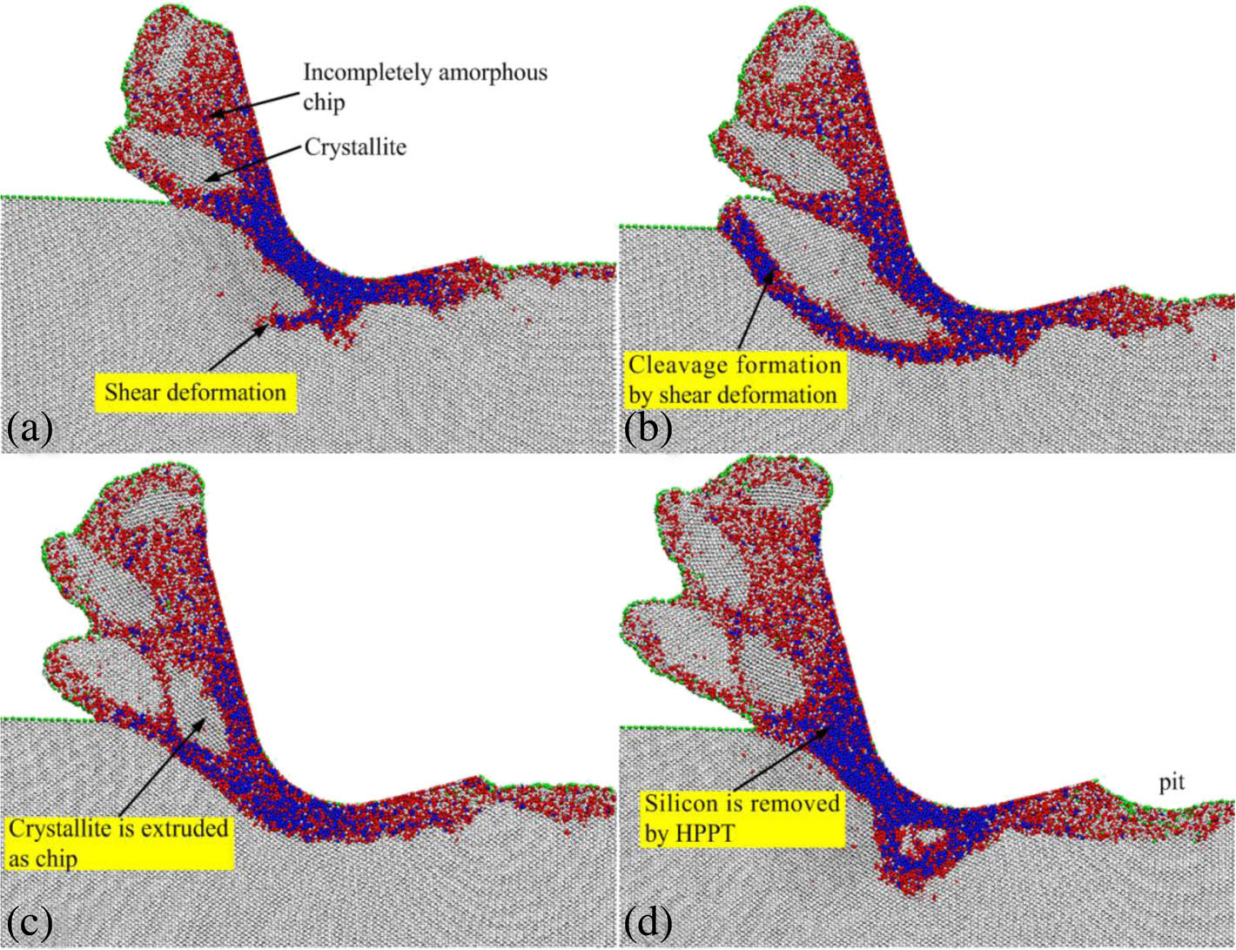
Sectional views on the (110)[00–1] direction at cutting distances of **a** 42 nm, **b** 46 nm, **c** 52 nm, and **d** 64 nm

As shown in Fig. 7, when cutting silicon in the (110)[00–1] crystal direction, the cutting forces have severe fluctuation. Particularly, the thrust force fluctuates with a maximum amplitude of about 550 nN, at a cutting distance of around 52 nm. To clearly illustrate the microscopic details during the fluctuation of cutting forces, A, B, C and D points are denoted in the curve of the thrust force in the figure, which correspond to the positions at cutting distances of 40, 46, 52, and 64 nm, respectively. Figure 8 shows the sectional views of the silicon workpiece in the cutting process. It can be seen in Fig. 8a, b that a shear deformation extends from the HPPT zone and finally reaches to the forward untreated surface, resulting in the cleavage occurrence and formation of a pretty large crystallite in the chip formation zone. It can be known according to the foregoing that the shear deformation is formed under the action of high shear stress, which can induce the amorphous transformation of silicon, even if the hydrostatic pressure is lower than the critical pressure for the HPPT. The hydrostatic pressure is sustained by the cutting forces in nanometric cutting process, in which the thrust force is dominated. Therefore, during the period from A to B points, the hydrostatic pressure decreases with the formation of the shear deformation, which cause the drop of the cutting forces, and the drop amplitude of the thrust force is obviously higher than the tangential force. As shown in Fig. 8c, the crystallite is nearly extruded from the chip formation zone, and the most of it is retained as chip. Owing to the energy that the crystallite requires to be removed by extrusion much lower than by HPPT, the hydrostatic pressure continuously decreases from B to C points, resulting in the successive drop of the thrust force and the tangential force. As indicated in Fig. 8d, the residual crystallite has been extruded, and no new cleavage appears. Because the crystallite is very large, its removal from the chip formation zone leaves a pit on the machined surface. At the moment, the material removal mode has changed from cleavage to HPPT. In order to sustain enough hydrostatic pressure for HPPT, the thrust force and the tangential force start to increase gradually after C point. The above results show that during the nanometric cutting process of silicon, the change of material removal mode can cause the severe fluctuation of the cutting forces.

**Fig. 9:**
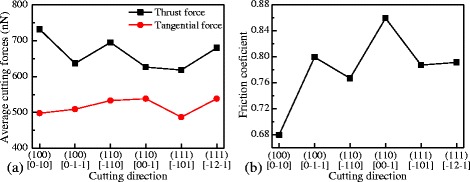
**a** Average cutting forces and **b** friction coefficient at the balanced phase

Figure 9 shows the average cutting forces and friction coefficient for different crystal directions at the stable cutting phase. It can be found in Fig. 9a that the thrust force is highest for the (100)[0–10] crystal direction and lowest for the (111)[−101] direction. From these values, the extent of anisotropic variation in the thrust forces is around 16%. Similarly, the tangential cutting forces vary by about 10%. As shown in Fig. 9b, the friction coefficient is the lowest for the (100)[0–10] crystal direction and the highest for the (110)[00–1] direction; the variation degree is about 21%.

## Conclusions

In this study, the effect of crystal anisotropy on cutting behaviors was explored through the large-scale MD simulations of nanometric cutting of single-crystal silicon in six different crystal directions. The following results were obtained:

1. The surface damage layer can be divided into the surface a-Si layer and SSD layer. The SSD is caused by shear deformation, and the orientation and complexity it forms are the primary causes that result in the SSD anisotropy. According to comparison of the surface integrity, it can be found that the (100) crystal plane is the best, while the (110) plane is the worst in nanometric cutting of silicon.
2. The chips on the (100)[0–10], (110)[−110], and (111) [−101] crystal directions are completely amorphous ones that are formed by HPPT, while the chips obtained by cutting along the (100)[0-1-1], (110)[00–1], and (111)[−12-1] directions are incompletely amorphous ones that contain large crystallites, which are the result of the combined action of cleavage and HPPT.
3. During nanometric cutting process of silicon, the change of material removal mode can cause the obvious fluctuation of the thrust force and the tangential force, and the amplitude of the former is much larger than the latter.
4. According to the material removal mode, the (100)[0–10], (110)[−110], and (111)[−101] crystal directions, compared with the other direction on the same crystal plane, are found to be more suitable to be removed in ductile mode. Moreover, through a comprehensive analysis on material removal mode, surface integrity and friction coefficient, among the six crystal directions, the (100)[0–10] direction is the best and the (110)[00–1] direction is the worst for ductile cutting.

## Appendix

1 $$ \mathrm{Stress}\ \mathrm{tensor}=\left[\begin{array}{ccc}\hfill {\sigma}_{xx}\hfill & \hfill {\tau}_{xy}\hfill & \hfill {\tau}_{xz}\hfill \\ {}\hfill {\tau}_{xy}\hfill & \hfill {\sigma}_{yy}\hfill & \hfill {\tau}_{yz}\hfill \\ {}\hfill {\tau}_{xz}\hfill & \hfill {\tau}_{yz}\hfill & \hfill {\sigma}_{zz}\hfill \end{array}\right] $$

Invariants:

2 $$ {I}_1={\sigma}_{xx}+{\sigma}_{yy}+{\sigma}_{zz} $$

3 $$ {I}_2={\sigma}_{xx}{\sigma}_{yy}+{\sigma}_{xx}{\sigma}_{zz}+{\sigma}_{yy}{\sigma}_{zz}-{\tau}_{xy}^2-{\tau}_{xz}^2-{\tau}_{yz}^2 $$

4 $$ {I}_3={\sigma}_{xx}{\sigma}_{yy}{\sigma}_{zz}+2{\tau}_{xy}{\tau}_{xz}{\tau}_{yz}-{\tau}_{yz}^2{\sigma}_{xx}-{\tau}_{xz}^2{\sigma}_{yy}-{\tau}_{xy}^2{\sigma}_{zz} $$

5 $$ {A}_1=-{I}_1;{A}_2={I}_2;{A}_3=-{I}_3 $$

6 $$ Q=\frac{3{A}_2-{A}_1^2}{9} $$

7 $$ R=\frac{9{A}_1{A}_2-27{A}_3-2{A}_1^3}{54} $$

8 $$ D={Q}^3+{R}^2 $$

if *D* < 0 then as follows:

9 $$ \theta ={ \cos}^{-1}\frac{R}{\sqrt{-{Q}^3}} $$

10 $$ {R}_1=2\sqrt{- Q}\times \cos \frac{\theta}{3}-\frac{A_1}{3} $$

11 $$ {R}_2=2\sqrt{- Q}\times \cos \frac{\theta +4\pi}{3}-\frac{A_1}{3} $$

12 $$ {R}_3=2\sqrt{- Q}\times \cos \frac{\theta +2\pi}{3}-\frac{A_1}{3} $$

13 $$ \begin{array}{l}\mathrm{Maximum}\ \mathrm{principal}\ \mathrm{stress}\left({\sigma}_1\right)= \max \left({R}_1,{R}_2,{R}_3\right);\\ {}\mathrm{Minimum}\ \mathrm{principal}\ \mathrm{stress}\left({\sigma}_3\right)= \min \left({R}_1,{R}_2,{R}_3\right)\end{array} $$

14 $$ {\sigma}_{\mathrm{hydrostatic}}=\frac{\sigma_{xx}+{\sigma}_{yy}+{\sigma}_{zz}}{3} $$

15 $$ {\sigma}_{\mathrm{Maximum}\ \mathrm{shear}\ \mathrm{stress}}=\frac{\sigma_1-{\sigma}_3}{2} $$
